# The role of amino bisphosphonates zoledronate and alendronate in shaping immunomodulatory profile and angiogenic potential of human periodontal ligament stem cells

**DOI:** 10.1371/journal.pone.0335744

**Published:** 2025-12-01

**Authors:** Jessica Bria, Emanuela Chiarella, Jessica Sovereto, Francesco Baudi, Marta Greco, Daniela Patrizia Foti, Amerigo Giudice, Anna Di Vito

**Affiliations:** 1 Department of Experimental and Clinical Medicine, University Magna Græcia, Catanzaro, Italy; 2 Department of Medical and Surgical Sciences, University Magna Graecia, Catanzaro, Italy; 3 Obstetrics and Gynecology Unit, University Hospital Renato Dulbecco of Catanzaro, Catanzaro, Italy; 4 Department of Health Sciences, University Magna Græcia, Catanzaro, Italy; 5 Clinical Pathology Unit, University Hospital “Renato Dulbecco” of Catanzaro, Catanzaro, Italy; Polytechnic University of Marche, ITALY

## Abstract

Bisphosphonate-related osteonecrosis of the jaw is a significant complication arising from the use of nitrogen-containing bisphosphonates such as Zoledronate (ZOL) and Alendronate (ALN). Although the effects of amino bisphosphonates on bone cells are well established, their influence on human periodontal ligament stem cells (hPDLSCs) remains inadequately explored. This study aimed to evaluate the influence of clinically relevant doses of ZOL and ALN on hPDLSCs secretome composition and its involvement in vascular changes reported in BRONJ. hPDLSCs isolated from the impacted third molar were exposed to increasing concentrations of ZOL (1 μM, 1.5 μM, 2 μM) or ALN (2 μM, 5 μM, 10 μM) for five days. Cytokine assay was directed to measure the levels of IL-1α, IL-6, IL-8, VEGF, and MCP-1. PD-L1 quantification was conducted by immunofluorescence, western blotting and flow cytometry. The angiogenic potential of hPDLSCs-derived secretome after preconditioning with ZOL or ALN was assessed on endothelial cells proliferation, migration, and tube-forming ability. Both ZOL and ALN treatment of hPDLSCs did not result in any significant changes in the secretion of inflammatory markers (IL-1α, IL-6, IL-8, VEGF and MCP-1). ZOL 1.5 μM (*p* < 0.0001) and 2 μM (*p* < 0.05) induced a down-regulation of total PD-L1. ALN 2 μM and 5 μM promoted a reduction of total (*p* < 0.0001) and surface (*p* < 0.01) PD-L1, whereas ALN 10 μM induced a rise in surface PD-L1 (*p* < 0.0001). Conditioned medium from ZOL- or ALN-treated hPDLSCs impairs cell migration (*p* < 0.05) and tube forming ability *in vitro* (*p* < 0.05). In summary, these results suggest that therapeutic concentrations of ZOL and ALN influence the immunomodulatory ability of hPDLSCs by altering PD-L1 expression and reducing the angiogenic potential of hPDLSCs secretome. Both the modulation of inflammation and impairment of angiogenesis may induce unfavorable conditions in periodontal tissue, facilitating the development of osteonecrosis.

## Introduction

Among the different types of osteonecrosis of the jaw (ONJ), bisphosphonates-related ONJ (BRONJ) stands out as a distinct form resulting from the use of bisphosphonates (BPs). Nitrogen-containing bisphosphonates (N-BPs) display a higher affinity for calcium than non-N-BPs and are rapidly taken up from circulation and bound to hydroxyapatite [1]. BPs are then released into the resorption lacunae, where they contribute to the pathogenesis of BRONJ. Many events have been associated with the development of necrosis, such as an imbalance between bone resorption and formation, infection and/or inflammation, immunosuppression, soft tissue toxicity, inhibition of angiogenesis and vitamin D deficiency [2–6].

Most of these hypotheses rely on the direct action of BPs on various cell types within the oral cavity, including osteoblasts, osteoclasts, and osteocytes in the bone, keratinocytes and fibroblasts in the oral mucosa, as well as immune and endothelial cells [7–10]. The anti-angiogenic effects of N-BPs have been recently described in both *in vivo* and *in vitro* studies [11–13]. N-BPs inhibit proliferation, adhesion and migration of endothelial cells [14,15]. Both non-N-BPs and N-BPs reduce the number of circulating endothelial progenitors as well as the endothelial differentiation from multipotent stem cells [16–17].

In the oral cavity, various sources of mesenchymal stem cells (MSCs) have been identified as effective and safe candidates for tissue regeneration [18]. Similar to MSCs isolated from different sources, dental MSCs have been characterized based on the ‘gold standard’ criteria established for MSCs from bone marrow (BMMSCs). However, oral MSCs display exclusive properties compared to MSCs of somatic origin, including a more pronounced commitment to odontogenic rather than osteogenic development [19,20]. Human periodontal ligament stem cells (hPDLSCs) are resident MSC of periodontal tissue which might be involved in periodontal ligament (PDL) regeneration, tissue homeostasis, and local inflammatory processes [21,22]. These events involve both the activation of hPDLSCs, altered cell proliferation and differentiation as well as the release of growth factors, cytokines, and chemokines which regulate the inflammatory microenvironment by modulating immune cells [22,23].

Many studies revealed that the biological and immunoregulatory properties of oral MSCs are compromised in BPs-treated patients, in agreement with *in vitro* studies showing a dose-dependent reduction of the proliferative and regenerative abilities of cells [24–27]. These events are responsible for the resorption-generation imbalance and infection reported during BRONJ pathogenesis. In particular, the increased apoptosis and decreased osteogenic ability of hPDLSCs could trigger periodontal disease, thus contributing to the onset of osteonecrosis of the jaw [25]. Recent research investigated the molecular mechanisms involved in such events, suggesting *PARP1* and *CYLD* as the two key genes involved in BPs-mediated inflammatory reactions in hPDLSCs [28]. However, the effects of BPs on the hPDLSCs-derived secretome and the immunoregulatory function remain poorly investigated. Immunomodulation is considered as the major mechanism of hPDLSCs therapeutic effect, and mostly involved factors include indoleamine-2,3-dioxygenase 1 (IDO-1), prostaglandin E2 (PGE2), tumor necrosis factor-inducible gene 6 protein (TSG-6), programmed cell death 1 ligand 1 (PD-L1), and programmed cell death 1 ligand 2 (PD-L2) [29]. PD-1/PD-L1 axis is a widely studied checkpoint that regulates immune responses [30]. PD-1 is primarily expressed on T cells, where it interacts with PD-L1 on antigen-presenting cells promoting immune suppression. In mesenchymal cells, the overexpression of PD-L1 is vital for the inhibition of inflammation. Despite the involvement of this checkpoint in bone health needs further investigations, a recent study showed that the inhibition of the PD1/PD-L1 signaling interferes with bone turnover and exerts a protective effect on bone by indirectly promoting osteogenesis [31].

Our hypothesis is that BPs modulate the immunophenotype of hPDLSCs and the proangiogenic role of their secretome, influencing wound healing properties and thus contributing to the onset of osteonecrosis of the jaw. Therefore, the main aim of the present study was to directly compare the effects of two potent N-BPs, Zoledronate (ZOL) and Alendronate (ALN), on the immunological properties of hPDLSCs. To elucidate how hPDLSCs immunomodulatory abilities can be affected by BPs at clinically relevant doses, we conducted a secretory analysis of BPs-treated hPDLSCs focusing on IL-1α, IL-6, IL-8, VEGF, and MCP-1. Additionally, to investigate the involvement of PD-L1 in hPDLSCs response, the expression of PD-L1 protein was evaluated after BPs-treatment. Hence, we directly compared the influence of conditioned media (CM) derived from BPs-treated hPDLSCs on the proliferation, migration, immunophenotype, and angiogenic properties of human umbilical vein endothelial cells (HUVECs). In this study, we described for the first time that the secretome derived from ZOL- and ALN-treated hPDLSCs negatively regulates the angiogenic potential of endothelial cells.

## Materials and methods

### Periodontal ligament stem cells isolation

The study was approved by the Regional Ethical Review Board of Central Calabria (reference for the Magna Graecia University of Catanzaro, Italy) (n. 268/2017). After obtaining written informed consent from all patients, PDL tissues were isolated from impacted third molars of six patients aged 19–25 years, recruited at the Unit of Oral Surgery—Academic Hospital of Magna Graecia University of Catanzaro from November 1^st^, 2017, to December 31^st^, 2018. hPDLSCs were isolated as reported in Di Vito et al. (2019) [32]. The cells were cultured for 7–14 days in 5% CO_2_ at 37°C to reach to 70%–80% confluency and then were subcultured as the first-cell passage in MesenPRO RS Medium (no. 12746012; Gibco, Life Technologies). The medium was changed every 3–4 days, and cells were used for further assays between the third and seventh passage. For each experiment, cells derived from at least three different individuals were used.

### Pharmacological treatment and collection of conditioned medium

hPDLSCs were seeded at a density of 4x10^3^ cell/cm^2^ on 60 mm plastic dishes. Upon reaching 60% confluency, the hPDLSCs were washed 3 times with PBS and then cultured for five days in fresh and complete MesenPro RS Medium added with ALN (1012780, Sigma, Milan, Italy) dissolved in sterile water, or ZOL (PHR1893, Sigma, Milan, Italy) dissolved in 0.1 N NaOH (S5881, Sigma, Milan, Italy). Cells cultured in the absence of the drugs were used as control. The chronic 5-day exposure used for this study was selected from our previous studies, where incubations longer than five days without replenishing the media significantly reduced the viability of hPDLSCs. The micromolar concentrations used in this study were selected from a wide range of previous studies on the concentrations of these drugs found in the serum of patients treated for osteoporosis or bone cancer after drug infusions [33–35]. In detail, the recommended dose of ZOL to prevent bone metastases in cancer patients is 4 mg every 4 weeks, achieving plasma concentrations of 1 μM in adults [34–36]. However, the concentration of ZOL in the bone tissue will be higher due to its tendency to accumulate in the bone matrix. Furthermore, a dose of 1 µM ZOL was described as the minimum concentration required to induce a significant inhibition of osteoclast formation i*n vitro* [37]. Therefore, in this study, ZOL was used at concentrations of 1 μM, 1.5 μM, and 2 μM, in order to recapitulate the *in vivo* conditions in the bone niche. As far as ALN is concerned, recommended dose in the treatment of postmenopausal osteoporosis is one 70 mg tablet once weekly. Plasma concentrations of ALN following therapeutic oral doses have been found to be less than 1 μM [33,38]. However, several studies have indicated that higher ALN concentrations are required to observe inhibitory effects *in vitro*. Accordingly, 10 μM ALN is required to achieve 50% inhibition of osteoclast activity *in vitro* [39], while 20 µM ALN is required to induce a decline in osteoblast proliferation [40]. Given that *in vitro* ALN concentrations ≤5 μM has been suggested as relevant for *in vitro* investigations, in this study ALN was used at concentrations of 2 μM, 5μM, and 10 μM. Conditioned medium was then collected, centrifuged at 2500 × g to remove cell debris, and stored at −80 °C until further use. Analysis was performed in cells deriving from at least three different donors.

### Cytokine assay

The “Cytokine Array I” kit (Randox Labs) was used according to the manufacturer’s guidelines to quantify the levels of cytokines in hPDLSCs-conditioned media collected after five days of exposure to the drug ZOL and ALN. Samples from non-treated and drug-treated cells were analyzed for the simultaneous quantification of the following cytokines: IL-1α, IL-6, IL-8, vascular endothelial growth factor (VEGF), and monocyte chemoattractant protein-1 (MCP-1). Conditioned medium from untreated hPDLSCs was used as control. Fresh medium was used as internal control for background measurement.

### Immunofluorescence analysis

Immunofluorescence was performed as previously described with some improvements [27]. hPDLSCs were exposed to increasing concentrations of ZOL (1 μM, 1.5 μM, and 2 μM) or ALN (2 μM, 5μM, and 10 μM) for five days. The cells were washed twice with PBS1X and then fixed with 0.3% glutaraldeyde (G5882, Sigma, Milan, Italy) for 10 min. hPDLSCs were then treated with 0.1% Triton X-100 (T8787, Sigma, Milan, Italy) at room temperature for 5 min and then with 1% bovine serum albumin (BSA, 05470, Sigma, Milan, Italy) for 30 min in order to block the nonspecific binding sites. Treated and untreated cells were incubated with anti-PD-L1 (no. PA5–28115; 1:200, rabbit; ThermoFisher Scientific) for 16 h (4°C) and then with Alexa-Fluor 488-conjugated anti-rabbit (1:500; Life Technologies) for 60 min at room temperature. The cells were counterstained with DAPI. All the samples were mounted using a fluorescent mounting medium. Images were acquired at 40X with a Leica fluorescence microscope (Leica DM IL LED, Leica Microsystems, Milan, Italy) and analyzed with ImageJ software (NIH Image Bethesda, MD). In order to quantify nuclear accumulation of PD-L1 from digital images, a specific ROI was set to measure the mean fluorescent intensity (MFI) in nucleus from individual cells. More than 55 cells were analyzed for each sample. The analysis was performed in triplicate, and at least four fields were acquired for each replicate.

### Total protein extraction and western blotting

Protein extraction and Western Blot were performed as previously reported by Di Vito et al. [27] Equal amounts of protein (40 μg) from untreated cells and cells exposed to increasing concentrations of ZOL (1 μM, 1.5 μM, and 2 μM) or ALN (2 μM, 5μM, and 10 μM) were loaded and separated by SDS-PAGE on 12% gels and transferred onto nitrocellulose membranes at 50V for 2 hours. (BioRad). Membranes were blocked for 1 hour at room temperature in PBS containing 0.05% (v/v) Tween-20 (PBST) and 5% dried milk or BSA and then incubated overnight at 4 °C with diluted primary antibodies, and specifically PD-L1 (1:1000, PA5–28115, ThermoFisher Scientific) and GAPDH (1:1000, sc-166574 Santa Cruz Biotechnology). The specificity of PD-L1 antibody has been previously reported [41]. After primary antibody incubation, membranes were washed three times with TBST for 10 min each, followed by incubation with a horseradish peroxidase-conjugated secondary antibody (Dako – Agilent Technologies). The expression of target protein was determined using an enhanced chemiluminescence system and then analyzed by ImageJ software, with GAPDH serving as an internal control for protein loading.

### Cell culture and treatment of human umbilical vein endothelial cells (HUVECs)

Human umbilical vein endothelial cells (HUVECs) were obtained from the American Type Culture Collection (ATCC) (Manassas, VA, USA) and were cultured in Endothelial Cell Medium (ECM) (1001-b-sc, CliniSciences, Italy) with 5% fetal bovine serum (FBS, Cat. No. 0025, CliniSciences, Italy), 1% of endothelial cell growth supplement (ECGS, Cat. No. 1052, CliniSciences, Italy) and 1% penicillin and streptomycin (Cat No. 0503, CliniSciences, Italy) in a 37°C humidified atmosphere containing 95% air and 5% CO_2_. The medium was changed every 3–4 days, and cells were used for further assays at passages three to seven.

For subsequent analysis, cells were organized in groups as follows: HUVECs exposed to conditioned medium (CM) collected from untreated hPDLSCs; HUVECs treated with CM collected from hPDLSCs exposed to increasing concentrations of ZOL (1 μM, 1.5 μM, and 2 μM) or ALN (2 µM, 5 µM, and 10 µM) for 5 days. The collected media were combined with complete ECM at a 50% concentration (50% CM + 50% complete ECM), and HUVECs were incubated with this mixture for different periods according to the subsequent experimental procedures.

### MTT assay

The MTT method was applied to evaluate the viability of HUVECs after exposure to CM collected from hPDLSCs exposed to increasing concentrations of ZOL or ALN. Briefly, cells (5 × 10^2^) were seeded into 96-well plates (Eppendorf, Milan, Italy) in 100 μL culture medium per well, prepared by combining ECM with CM of non-treated hPDLSCs and ZOL- or ALN-treated hPDLSCs at a 50% (v/v) concentration. All experimental conditions were tested in 8 replicates. After 24, 48, 72 and 96 h of treatment, 10 μL of MTT solution (5 mg/mL; M2128, Sigma, Milan, Italy) was added to each well. The plates were incubated at 37 °C for 2.5 h and then the supernatant was removed. Formazan crystals were solubilized by adding 0.08 N HCl (H1758, Sigma, Milan, Italy) in isopropanol (I9516, Sigma, Milan, Italy) for 20 min at room temperature (RT). The absorbance was measured at 595 nm using a microplate reader (BioTek 800 TS Absorbance Reader, Agilent). Values obtained in the absence of cells were considered as internal control for background measurement. Assay was performed three times on biological replicates.

### Flow cytometry

To assess PD-L1 expressions on hPDLSCs exposed to ZOL and ALN, indirect labeling was conducted. Briefly, 1 × 10^5^ cells were incubated with anti-PD-L1 antibody (PA5–28115, ThermoFisher Scientific) for 30 minutes at 4°C, followed by three washes with cold PBS. Subsequently, the binding between the antibody and antigen was revealed by incubating the cells with a secondary antibody directly conjugated to Alexa-Fluor 488 (A11070) fluorochrome for 30 minutes at 4°C in the dark.

The immunophenotype of HUVECs exposed to CM from non-treated and ZOL- or ALN-treated hPDLSCs was assessed after five days, following the protocol outlined in Chiarella et al [42]. Briefly, 1 × 10^5^ cells were stained with PE-conjugated anti-CD34 (clone AC136; Miltenyi Biotec, Bergisch Gladbach, Germany), FITC-conjugated anti-CD105 (clone 43A4E1, Miltenyi Biotec) and FITC-conjugated anti-CD31 (clone WM59, BD) for 30 minutes at 4°C in the dark.

After staining, the cells were washed twice with cold PBS and resuspended in 300 µL of PBS for acquisition on a BD FACSCanto II flow cytometer. Ten thousand events were recorded from each sample. The analysis was performed using FlowJo software version 8.8.6, employing an optimal gating strategy to select viable cells while excluding dead cells, which could interfere with accurate signal analysis due to high autofluorescence levels and non-specific antibody binding. The threshold to define PD-L1 + , CD34 + , CD31 + , and CD105 + cells was established using a negative control (sample without antibody). The experiment was performed three times.

### Scratch wound healing assay

A total of 2 × 10^5^ HUVECs were seeded into 6-well plates and cultured until confluency. The monolayers were scratched with a 200 µL pipette tip and washed three times with PBS. Then, 1 mL of medium (50% CM + 50% complete ECM) was added. Only wells with a uniform initial scratch size were used in the assay to provide the correct normalization. The wounded monolayers were observed at 20X under a phase contrast inverted microscope (Leica DM IL LED, Leica Microsystems, Milan, Italy) and photographs were taken immediately after scratching and again 6 hours later.

Areas of wound closure were determined manually by means of ImageJ Version 1.50i software, and relative wound areas were calculated as (wound area at 6 h)/(initial wound area) ratio. For each experimental condition studied, the scratch assay was performed in at least four biologically independent experiments. At least five fields for each replicate were collected.

### Tube formation assay

A drop of 50 μL of pre-thawed Matrigel matrix (Corning Inc., Corning) was used to coat a pre-chilled 48-wells plate and allowed to polymerize at 37 °C for 30 min. Then, 3,75 × 10^4^ HUVECs were seeded onto the solidified Matrigel matrix in CM of ZOL- or ALN-treated PDLSCs (50% v/v). Four and twenty-two hours later, the wells were photographed and the number of nodes, meshes, and segments as well as total segments length were analyzed using the ImageJ angiogenesis analyzer. At least three representative fields per well were captured using phase contrast microscopy. The experiment was performed three times.

### Statistical analysis

Three healthy patients were included in this study. Data are expressed as mean ± standard deviation (S.D.). In alternative, data from a representative experiment are shown. GraphPad Prism version 8.0.1. was used for statistical analysis. Before applying parametric tests, the normality of data distribution was assessed using the Shapiro-Wilk test or D’Agostino & Pearson test. One-way ANOVA followed by the Tukey post hoc test was used for determination of statistical differences. p < 0.05 was considered statistically significant and the number of replicates was indicated in each experiment. Figures include error bars to represent S.D., and significant differences are highlighted using asterisks. For the analysis performed on HUVECs, significant differences versus CM(-drug) were indicated by asterisk, while statistically significant differences versus Endothelial Cell Medium (ECM) were indicated by hash as follows: *,^#^, p < 0.05, **,^##^, p < 0.01, ***,^###^, p < 0.001, ****,^####^, p < 0.0001.

## Results

### Effects of ZOL or ALN on the Expression of Immunomodulatory Proteins in hPDLSCs

#### Cytokines secretion in ZOL- or ALN-treated hPDLSCs cultures.

**Fig 1:**
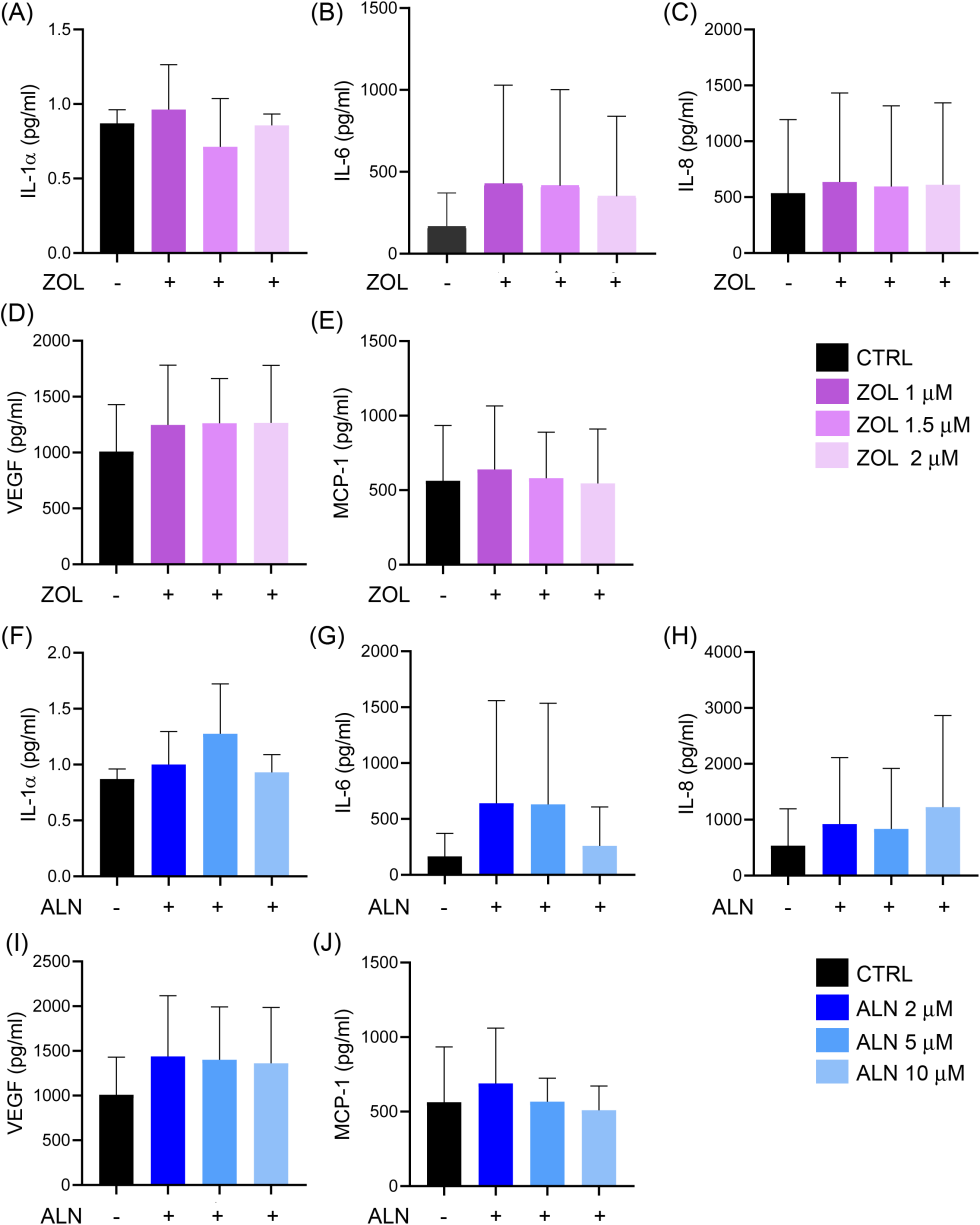
Cytokines analysis. The levels of interleukin-1α (IL-1α), interleukin-6 (IL-6), interleukin-8 (IL-8), vascular endothelial growth factor (VEGF), and monocyte chemoattractant protein-1 (MCP-1) were measured in the culture media from human periodontal ligament stem cells (hPDLSCs). The cells were treated with zoledronate (ZOL) at concentrations of 1 μM, 1.5 μM, or 2 μM (A-E) or with alendronate (ALN) at concentrations of 2 μM, 5 μM, or 10 μM (F-J). Data are expressed in (pg/mL) ± S.D; *n* = 4 independent experiments. After exposure to ZOL: IL-1α, *p* = 0.3440; IL-6, *p* = 0.8499; IL-8, *p =* 0.9973; VEGF, *p =* 0.8134; MCP-1, *p* = 0.9827. After exposure to ALN: IL-1α, *p* = 0.1152; IL-6, *p* = 0.6253; IL-8, *p =* 0.8569; VEGF, *p =* 0.6833; MCP-1, *p* = 0.8113.

We evaluated the production of cytokines that could be involved in the immunoregulatory response of hPDLSCs after ZOL or ALN exposure. The concentration of cytokines detected in the CM of hPDLSCs cultured in the absence of drugs were considered as control. The concentrations of IL-1α, IL-6, IL-8, VEGF, and MCP-1 were determined in all CM of hPDLSCs exposed to drugs (Fig 1). After five days of treatment with ZOL or ALN no significant changes were reported in secretion of inflammatory markers (*p* = 0.1152−0.9973; Fig 1A-1J).

#### PD-L1 Expression in ZOL- or ALN-treated hPDLSCs cultures.

**Fig 2:**
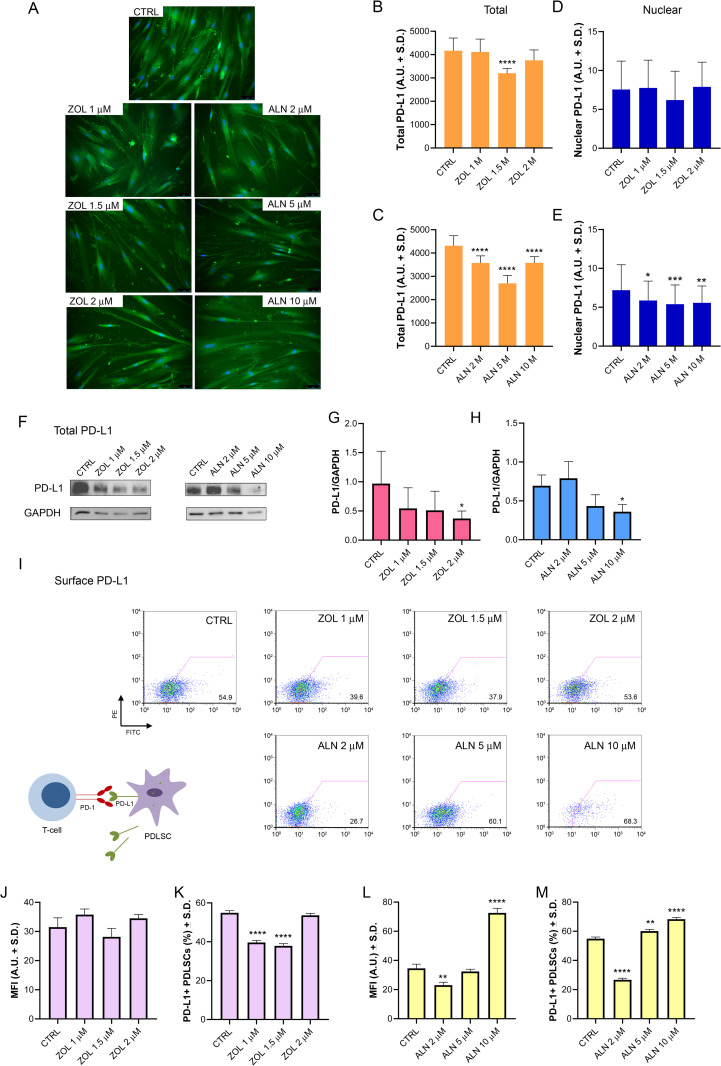
PD-L1 expression in hPDLSCs exposed to ZOL or ALN. (A) Immunofluorescence analysis of PD-L1 (green) in hPDLSCs untreated and treated with ZOL or ALN for 5 days. Nuclei are indicated in blue. (B-E) ImageJ analysis of the expression of total and nuclear PD-L1. (F) Western blot results for PD-L1 expression. GAPDH was used for normalization. Original blots are presented in Supplementary File 1 (S1-S8 Fig3). (G, H) Quantitative comparison of PD-L1 expressions in hPDLSCs by western blot. (I) Flow cytometry results of PD-L1 surface expression in hPDLSCs. (J-M) Quantitative comparison of the percentage of PD-L1 + cells and PD-L1 Mean Fluorescence Intensity (MFI) in hPDLSCs treated or untreated with ZOL or ALN. MFI reflects the average level of fluorescence (protein expression) *per* cell, while the percentage of positive cells indicates the proportion of cells that exhibit a detectable level of fluorescence above background. Data are shown and expressed as arbitrary units (A.U.) ± S.D. (*n* = 3 independent experiments). Significant differences versus untreated cells (CTRL) were indicated by asterisk as follows: *, p < 0.05; **, p < 0.01; ***, p < 0.001; ****, p < 0.0001.

We showed that exposure to increasing concentrations of ZOL and ALN modulated PD-L1 protein expression in hPDLSCs (Fig 2). Immunofluorescence analysis indicated a very strong down-regulation of PD-L1 after exposure to ZOL 1.5 μM compared to untreated cells (*p* < 0.0001; Fig 2A, 2B). Western blot analysis confirmed a significant reduction of PD-L1 after exposure to ZOL 2 μM (*p* = 0.0168; Fig 2F, 2G). No significant variations were observed on surface PD-L1 after ZOL exposure (*p* = 0.2240; Fig 2I, 2J). Additionally, exposure to ZOL 1 μM and 1.5 μM accounted for a significant reduction in the percentage of PD-L1 + cells respect to untreated cells (p < 0.0001; Fig 2I, 2K).

Treatment with increasing concentrations of ALN determined a spatial reorganization of PD-L1. Immunofluorescence analysis showed a significant down-regulation of total PD-L1 in the presence of ALN at different concentrations (*p* < 0.0001), also confirmed by western blot at the highest ALN dose 10 μM (*p* = 0.0427; Fig 2A, 2C, 2F, 2H). Interestingly, ALN 2 μM induced a significant reduction in both nuclear (*p* = 0.0364) and surface (*p* = 0.0021) PD-L1 (Fig 2A, 2E, 2L), while the exposure to ALN 5 μM determined a significant reduction of nuclear PD-L1 (*p* = 0.0010) while did not change the expression of surface PD-L1 (p = 0.7358; Fig 2E, 2L). Interestingly, ALN 10 μM induced a significant reduction of nuclear PD-L1 (p = 0.0022) and a strong overexpression (*p* < 0.0001) of surface PD-L1 (Fig 2E, 2L), suggesting a spatial redistribution of the protein. Moreover, low concentrations of ALN determined a significant reduction (*p* < 0.0001) in the percentage of PD-L1 + cells, whereas higher concentrations (5 μM and 10 μM) determined a significant increase (*p* = 0.0018 and *p* < 0.00001, respectively) in the number of cells expressing PD-L1 (Fig 2M).

### Effect of preconditioning hPDLSCs with ZOL or ALN on the angiogenic potential of hPDLSCs-secretome

#### ZOL- and ALN-Conditioned hPDLSC Media did not modulate HUVECs Proliferation.

**Fig 3:**
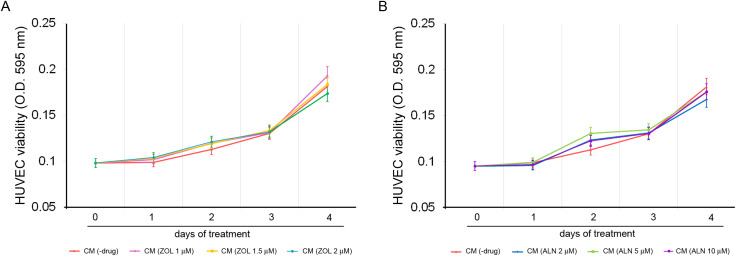
Cell viability of HUVECs after incubation with CM from (A) ZOL- or (B) ALN-treated hPDLSCs. Data are presented as the mean ± S.D. (*n* = 3 independent experiments). In each analysis, a *p*-value < 0.05 was considered statistically significant.

Angiogenesis involves multiple processes, including the proliferation, migration, and differentiation of endothelial cells. We evaluated the angiogenic potential of the secretome released by BPs-treated hPDLSCs using HUVECs. Although HUVECs proliferation increased after 24, 48, and 72 h of incubation with CM from ZOL- (CM(ZOL)) or ALN- (CM(ALN)) treated hPDLSCs compared to CM from untreated cells (-drug) (Fig 3A, 3B), statistical analysis did not show significant differences between the experimental conditions (*p* = 0.4254–0.9760 in CM(ZOL)-treated HUVECs; *p* = 0.1493–0.9760 in CM(ALN)-treated HUVECs).

#### ZOL- and ALN-Conditioned hPDLSCs Media did not modulate HUVECs Phenotype.

**Fig 4:**
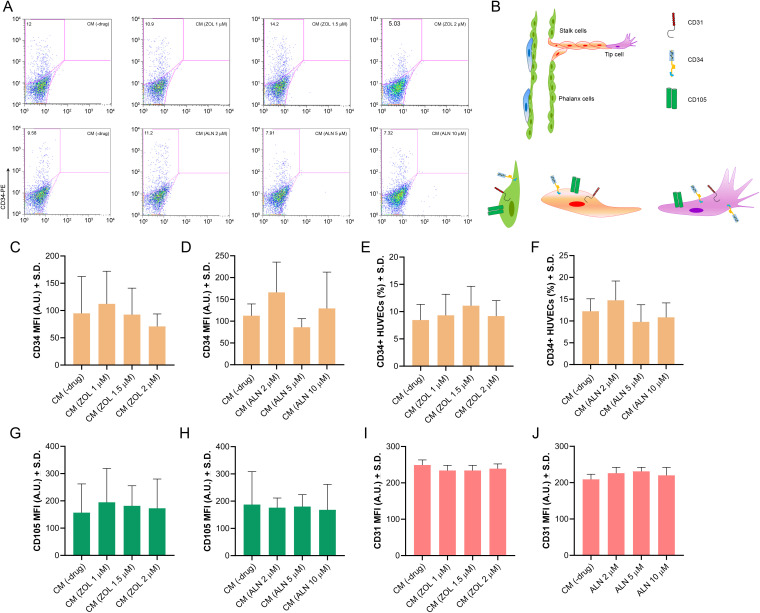
Effect of CM from ZOL- or ALN-treated hPDLSCs on the endothelial phenotype of HUVECs. (A) Representative flow cytometry histograms showing CD34 expression in HUVECs treated with CM from untreated hPDLSCs (CM(-drug)) or hPDLSCs treated with ZOL (1 µM, 1.5 µM, or 2 µM) or ALN (2 µM, 5 µM, or 10 µM). (B) Schematic diagram illustrating endothelial cell phenotypes (tip, stalk, and phalanx cells) based on CD31, CD34, and CD105 expression. (C, D) Quantitative comparison of mean fluorescence intensity (MFI) for CD34 in HUVECs treated with CM(ZOL) (C) or CM(ALN) (D). (E, F) Percentage of CD34 + HUVECs after treatment with CM(ZOL) (E) or CM(ALN) (F). (G-J) MFI values for CD105 (G, H) and CD31 (I, J) in HUVECs treated with CM(ZOL) or CM(ALN). Data from a representative experiment are shown and expressed as (MFI) or (%) ± S.D. (*n* = 3 independent experiments). p < 0.05 indicates significant differences compared to control.

The influence of CM(ZOL) or CM(ALN) on the phenotype of HUVECs was investigated by flow cytometry and compared to CM(-drug) (Fig 4). CD34 plays key roles in endothelial cell activity. CD34 + HUVECs have been compared to sprouting tip cells, which are specialized cells at the extremity of newly formed capillaries [43]. Moreover, CD34 and CD105 are overexpressed on proliferating endothelial cells *in vitro* [44,45]. Therefore, these markers were used to distinguish between endothelial tip cells (CD34+) and proliferating endothelial cells (CD105+) within the CD31 + endothelial population (Fig 4B).

Exposure of HUVECs to CM from ZOL- or ALN-treated hPDLSCs did not induce significant differences (*p* = 0.7456 in CM(ZOL)-treated cells, *p* = 0.4219 in CM(ALN)-treated cells) in CD34 expression between experimental groups (Fig 4A, 4C, 4D). Similarly, no significant alterations (*p* = 0.8660 in ZOL-treated cells, *p* = 0.9782 in ALN-treated cells) were reported in the percentage of CD34 + cells in CM(ZOL)- or CM(ALN)-treated HUVECs compared to cells exposed to CM(-drug) (Fig 4E, 4F). Further analysis showed that neither ZOL- nor ALN-treated CM significantly altered the surface expression of CD31 or CD105 in HUVECs compared to controls (*p* = 0.4308–0.9956; Fig 4G-4J).

#### ZOL- and ALN-Conditioned hPDLSC Media modulated HUVECs Migration.

**Fig 5:**
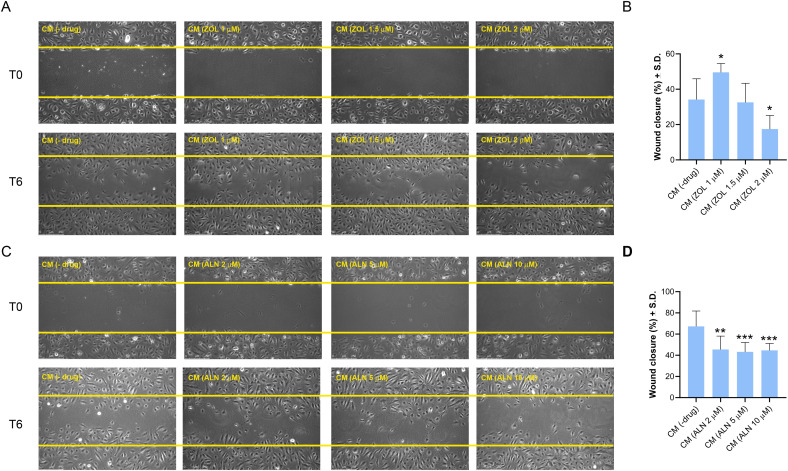
Analysis of the wound healing ability of HUVECs treated or untreated with CM(ZOL) or CM(ALN). (A, C) Cells were scratched and incubated with 1 mL of medium (50% CM + 50% complete ECM). HUVECs migration was visualized and recorded at 0 and 6 h post-scratch. The yellow line indicates the border of the wound. Scale bar 75 µm. The closed area was measured by NIH Image J and was calculated as the ratio of the remaining area at the 6-hr time point to the 0-hr starting point. (B) Graph of measurements in panel A. (D) Graph of measurements in panel C. Data from a representative experiment are shown and expressed as (%) ± S.D. (*n* = 3 independent experiments). In each analysis, a *p*-value < 0.05 was considered statistically significant. Significant differences versus CM(-drug)-treated cells were indicated by asterisk as follows *, p < 0.05; **, p < 0.01; ***, p < 0.001.

After 6 h of incubation with CM(ZOL 1 μM), HUVECs migration significantly increased compared to CM(-drug) (*p* = 0.0271) (Fig 5A, 5B). Conversely, HUVEC migration decreased with CM (ZOL 1.5 μM and 2 μM), with the reduction becoming statistically significant at the highest concentration (*p* = 0.0289). On the other hand, CM(ALN) reduced cell migration (Fig 5C, 5D) at all tested concentrations (*p* = 0.0021 for CM(ALN 2 μM); *p* = 0.0003 for CM(ALN 5 μM); *p* = 0.0003 for CM(ALN 10 μM)).

#### Effects of conditioned media from ZOL- or ALN-treated hPDLSCs on the angiogenesis process.

**Fig 6:**
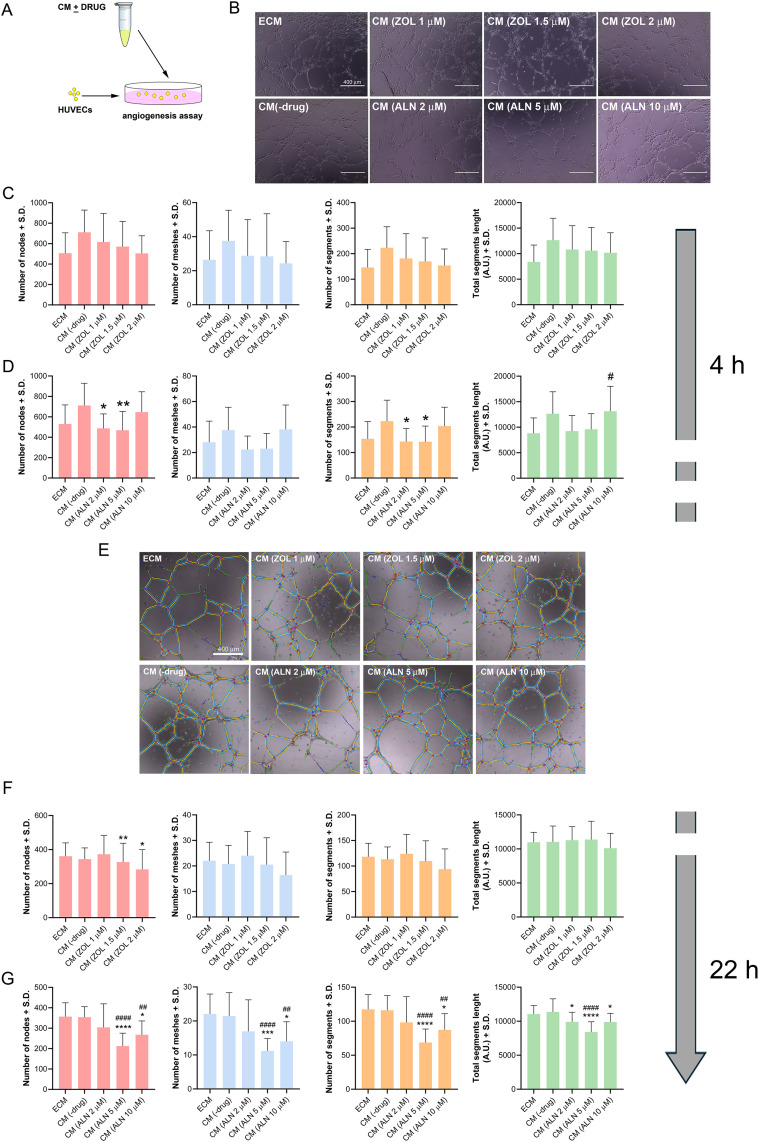
Results from angiogenic assay performed in the presence of CM(ZOL) or CM(ALN). (A) Figure shows the scheme of the experimental setup. (B, E) Representative microphotographs of tubulogenic assay were shown at 4 h and 22 h. Images have been analyzed with NIH ImageJ software and Angiogenesis analyzer plugin. (C, D, F, G) The graphs represent the estimation of tubulogenic potential of HUVECs exposed to CM(ZOL) (C, F) or CM(ALN) (D, G). Data have been obtained by analyzing the number of nodes (pixels with at least three neighboring elements corresponding to a bifurcation), meshes (areas enclosed by segments or master segments, made by tube-like-structures), segments (elements delimited by two junctions), and total segments length. Statistical analysis (4 h, *vs* CM(-drug)): number of nodes (*p* = 0.0161 for CM(ALN 2 μM); *p* = 0.0073 for CM(ALN 5 μM)); Number of segments (*p* = 0.0184 for CM(ALN 2 μM); *p* = 0.0168 for CM(ALN 5 μM)); Statistical analysis (22 h, *vs* CM(-drug)): Number of nodes (*p* = 0.0020 for CM(ZOL 1.5 μM); *p* = 0.0345 for CM(ZOL 2 μM); *p* < 0.0001 for CM(ALN 5 μM); *p* = 0.0123 for CM(ALN 10 μM)); Number of meshes (*p* = 0.0002 for CM(ALN 5 μM); *p* = 0.0111 for CM(ALN 10 μM)); Number of segments (*p* < 0.0001 for CM(ALN 5 μM); *p* = 0.0138 for CM(ALN 10 μM)); Total segments length (*p* = 0.0431 for CM(ALN 2 μM); *p* < 0.0001 for CM(ALN 5 μM); *p* = 0.0383 for CM(ALN 10 μM)). Data from a representative experiment are shown and expressed as (Number) or (A.U.) ± S.D. (*n* = 3 independent experiments). In each analysis, a *p*-value < 0.05 was considered statistically significant. Significant differences versus CM(-drug) were indicated by asterisk, while statistically significant differences versus Endothelial Cell Medium (ECM) were indicated by hash as follows: *,^#^, *p* < 0.05; **,^##^, *p* < 0.01; ***,^###^, *p* < 0.001; ****,^####^, p < 0.0001.

To investigate the effects of CM(ZOL) or CM(ALN) on the ability of HUVECs to form tubule networks *in vitro*, a tube formation assay was performed as reported in Fig 6A. The network of tubes formed was quantified with the NIH ImageJ analysis software after 4 and 22 h. Four indicators to determine the angiogenic effects of CM from BPs-treated hPDLSCs were analyzed: the number of nodes, the number of meshes, the number of segments, and total segments length. After 4 h, a non-significant trend toward increased tubulogenic activity was observed in cells exposed to CM(-drug) when compared to cells maintained in ECM, highlighting the angiogenic potential of MSCs-derived secretome. CM(ZOL 1 μM) had a neutral effect on angiogenesis (Fig 6B, 6C, 6E, 6F). Conversely, a gradual and significant reduction of the number of nodes was reported in a dose-dependent manner at the higher ZOL concentrations (*p* < 0.05) after 22 h. A significant anti-angiogenic action was reported in HUVECs exposed to CM(ALN) at 4 h and 22 h (Fig 6B, 6D, 6E, 6G). The number of nodes, the number of meshes, the number of segments, and total segments length were significantly lower (*p* < 0.05) in HUVECs exposed to CM(ALN) than in the CM(-drug)-treated HUVECs.

## Discussion

Reduced bone remodeling, delayed soft tissue healing, and aberrant angiogenesis contribute to the pathogenesis of BRONJ. These processes originate from the direct influence of BPs on multiple cell types within the periodontal niche, including osteoclasts, osteoblasts, osteocytes, fibroblasts, epithelial cells, mesenchymal stem cells, and endothelial cells [46]. In addition to their cell-specific effects, BPs also disrupt cell-cell communication and paracrine signaling within the context of BRONJ. During BPs therapy, the hPDLSCs undergo a “conditioning” that alters their regenerative potential as well as the secretome profile, impacting both the soluble protein fraction—comprising growth factors and cytokines—and the vesicular fraction. Based on these considerations, we point our attention to the effects of ZOL and ALN on the immunophenotype of hPDLSCs and the angiogenic potential of their secretome.

A first consideration in our experimental setup regards drug concentration. Our group and others showed that N-BPs, at clinically relevant doses, can slow cell proliferation and impair wound healing without inducing cell death [24,27,45]. The reduced cell growth observed in hPDLSCs cultures exposed to ZOL and ALN results from cell cycle arrest and a modulation of osteogenic differentiation. Conversely, cell death is typically observed only at the highest doses of ZOL and ALN [24,27]. Similarly, Giannasi et al., 2019 showed that high doses of both ZOL and ALN exerted a cytotoxic effect on osteoblastic cells, while lower doses affected the short-term release of several bone markers and cytokines [47]. For this study, we considered drugs concentration that closely resemble the *in vivo* ones. Accordingly, the serum concentration of ZOL is about 1–2 μM, while *in vitro* ALN concentrations ≤5 μM best mimic *in vivo* observations [40,48].

In our study, increasing concentrations of ZOL or ALN did not result in any significant changes in secretion of the inflammatory markers IL-1α, IL-6, IL-8, VEGF, and MCP-1. IL-1α, IL-6, IL-8, and MCP-1 are pro-inflammatory interleukins with a key role in promoting osteoclastogenesis and angiogenesis *in vivo*. The two isoforms of IL-1, IL-1α and IL-1β, modulate bone metabolism by promoting osteoclast differentiation and formation [49,50]. Conversely, IL-6 displays a dual role in bone metabolism, by activating bone resorption or bone formation depending on the specific microenvironment [51]. Both IL-8 and MCP-1 also promote osteoclastogenesis [52,53]. Previous studies reported an increased release of the pro-inflammatory cytokines IL-1α and IL-6 in fibroblasts, osteoblast- and osteocyte-like cells after ZOL treatment [54–56]. Notably, a concentration of 10 μM ZOL enhanced the production of proinflammatory cytokines by inducing M1 macrophage polarization [57]. Similarly, ALN was able to induce the release of pro-inflammatory cytokines by mouse macrophage-like cell lines [58–60]. VEGF is a signaling molecule that enhances endothelial cell survival, differentiation, and osteogenesis. Reduced VEGF levels in the serum of patients with MRONJ have been associated with decreased vascularity and reduced number of microvessels in the early stages of bone healing [61]. *In vitro* studies showed contradictory results. In the studies of Kruger et al., ALN 5 μM did not alter VEGF secretion, while ALN 20 μM decreased it by 50% [40]. Conversely, Ishtiaq et al. showed that ALN 1 μM was sufficient to decrease VEGF secretion in osteoblastic cell lines [62]. Manzano-Moreno et al. showed an increased expression of VEGF in osteoblasts after exposure to ALN concentrations ranging from 10 μM to 1 mM [63]. Another study reported that ALN 5 μM increased MCP-1 expression in osteoblasts after three days of incubation, while no effect was reported during longer treatments [40]. The high heterogeneity of the data obtained *in vitro* derives from the use of different cellular models, different drug concentrations and exposure times, confirming the need for standard condition to define the action of BPs.

The most important factors mediating hPDLSCs immunomodulatory activities include IDO-1, PGE2, TSG-6, PD-L1, and PD-L2 [29]. These immunomodulatory effects have been reported *in vitro* and/or *in vivo* on T cells, B cells, dendritic cells, macrophages and poly-morphonuclear neutrophils (PMNs). Among the different factors, PD-L1 mediates cell-to-cell contact mechanisms by which hPDLSCs negatively regulate the proliferation, differentiation and chemotaxis of activated B cells *in vitro* [64,65]. PD-L1 overexpression is recognized as key mechanism for suppressing CD4^+^ T lymphocyte proliferation by hPDLSCs [29]. Furthermore, PD-1/PD-L1 axis plays a key role in regulating γδT cells, which are strongly activated by phosphoantigens including isopentenyl pyrophosphate (IPP) [66]. The phosphoantigens can specifically activate Vγ9Vδ2 T cell subset at pico- to nanomolar concentrations. Given that exposure to N-BPs determines intracellular accumulation of IPP allowing γδ T cells to activate, we assumed that the regulation of PD-L1 by ZOL or ALN could modulate γδ T cells-mediated inflammation. In our study, the reduced PD-L1 expression observed in almost all experimental conditions suggests a deregulation of PD-L1/PD-1 axis. MSCs of the oral cavity express variable levels of PD-L1 in response to both microbiome diversity and health status of the people [29,67–70]. It’s noteworthy that PD-L1 can exert distinct biological effects depending on its cell localization (cell surface, cytosol, nucleus) or extracellular secretion [71]. Emerging studies have reported that PD-L1 translocates into the nucleus and plays a role in tumorigenesis, by promoting proliferation, metastasis, or immunotherapy resistance [72,73]. Recently it was also reported that nuclear PD-L1 can trigger angiogenesis through the regulation of gene expression in uveal melanoma [74]. We reported that exposure to ZOL 1 μM and ZOL 1.5 μM reduced the percentage of PD-L1 + cells, and ZOL 1.5 μM and 2 μM reduced total PD-L1 expression, suggesting the induction of a proinflammatory profile. Notably, preconditioning with ALN induced the major alterations. In this case, the exposure to the lower ALN concentration resulted in an overall downregulation of PD-L1, based on reduction in total, nuclear and surface PD-L1. However, significant upregulation of surface PD-L1 was reported with increasing drug concentrations, suggesting that ALN accumulation in the alveolar bone might be responsible for the stimulation of canonical PD-L1 function of T cell dysfunction. This observation well fits with the increased percentage of PD-L1 + cells reported at higher ALN concentrations. To date, the effects of BPs on the expression of the major factors mediating hPDLSCs immunomodulatory activities remain poorly investigated. ALN was reported to increase the expression of immune-related factors, including TGF-β1, NOS, PTGS, HGF, NO, PEG2, IDO, and IL-6 in adipose-derived stem cells, indicating in this case an anti-inflammatory action [75]. While the inhibition of the PD1/PD-L1 signaling pathway has been suggested to have a protective role in bone, a case of severe osteonecrosis of the jaw due to anti-PD-1 therapy has been recently described *in vivo*, suggesting the need for further analysis [76]. Furthermore, more in-depth investigations are required to understand non-canonical activities of cytosolic and nuclear PD-L1.

hPDLSCs-secretome plays a key role in angiogenesis by stimulating endothelial cells functions *in vitro* and vessel sprouting *in vivo* [77–79]. To this purpose, hPDLSCs release several angiogenic factors, including VEGF, FGF2, PDGF, PGF, IGF-I, stem cell factor (SCF), MCP-1 [80]. In our study, CM derived from ZOL- or ALN-treated hPDLSCs did not induce significant alterations in endothelial cell proliferation. Accordingly, the expressions of CD31 and CD105, with CD105 being a recognized marker of proliferating endothelial cells, remained unaffected by the treatments. Conversely, the secretome of ZOL or ALN-treated hPDLSCs modulated endothelial cells migration and angiogenesis. Endothelial cell migration is an essential component of vessel formation [81]. Furthermore, the arrangement of endothelial cells (EC) in tip and stalk cells has been recognized as the main driver of angiogenesis [82]. Our results showed a non-significant trend toward increased CD34 and capillary-like structures formation after incubation with CM(ZOL 1 μM), whereas a significant increase in endothelial cell migration was shown. Similarly, the non-significant dose-dependent reduction of CD34 expression after incubation with CM (ZOL 1.5 μM and 2 μM) well fit with the reduced cell migration observed in these experimental conditions. The anti-angiogenic action of CM(ZOL 1.5 μM) and CM(ZOL 2 μM) was also confirmed by a significant reduction of tube formation. How do we explain these apparently contradictory results? A possible explanation derived from the different cytokine concentrations detected in CM from hPDLSCs exposed to increasing ZOL concentrations. Indeed, the highest concentrations of cytokines and growth factors with a clear role in angiogenesis, such as VEGF, MCP-1 and IL-1ɑ were detected after exposure to ZOL at 1 μM. We are not surprised by such a result, as ZOL also performed a similar action against hPDLSCs [24].

A different trend was observed in HUVECs incubated with CM(ALN). Both angiogenic potential and migration of the cells were strongly reduced in HUVECs exposed to CM(ALN). CM(ALN 5μM) had the strongest inhibitory effects on the angiogenesis and was able to induce a non-significant trend towards reduced percentage of CD34 + cells and CD34 MFI.

In this study, we decided to continuously expose hPDLSCs to ZOL or ALN, to ensure an experimental design closer to *in vitro* testing applications and clinical practice. The pharmacokinetics and pharmacodynamics of ZOL and ALN are extremely complex. Evidence showed that BPs effects depend on the ability of cells to internalize enough N-BP to inhibit the mevalonate pathway [83]. After cellular uptake, the degradation rate of ZOL is considered negligible, as it is not metabolized; instead, cellular ZOL is only released back into the medium. Interestingly, both *in vivo* and *in vitro* studies confirmed that transfer rate from medium to tumor cells of ZOL is eight-fold higher than the transfer rate from the cell to the medium [84,85]. A similar pharmacodynamic can be assumed for ALN. Therefore, although the presence of residual N-BPs in the conditioned medium cannot be completely excluded, their impact on HUVEC tubulogenesis is likely to be minimal.

One key limitation of our study is the insufficient exploration of the molecular mechanisms underlying the effects of ZOL and ALN on hPDLSCs and endothelial cells. While we have observed important cellular responses *in vitro*, the specific molecular pathways driving these effects remain unclear. Future research should aim to delve deeper into these mechanisms by investigating relevant signaling pathways and exploring gene expression profiling. This will provide a more comprehensive understanding of how N-BPs influence the intricate balance between immune modulation and angiogenesis, which is crucial for developing targeted therapies. Additionally, while our *in vitro* findings are promising, they are limited in their ability to reflect the complex physiological conditions of a living organism. The lack of *in vivo* validation remains a significant challenge, as animal models are necessary to confirm the relevance and applicability of our results. For example, preclinical models of BRONJ or periodontal disease could be used in future studies to explore how BPs-treated hPDLSCs influence angiogenesis and tissue regeneration within a living system. This will help ensure that our observations are not only scientifically robust but also biologically and clinically relevant.

## Conclusions

**Fig 7:**
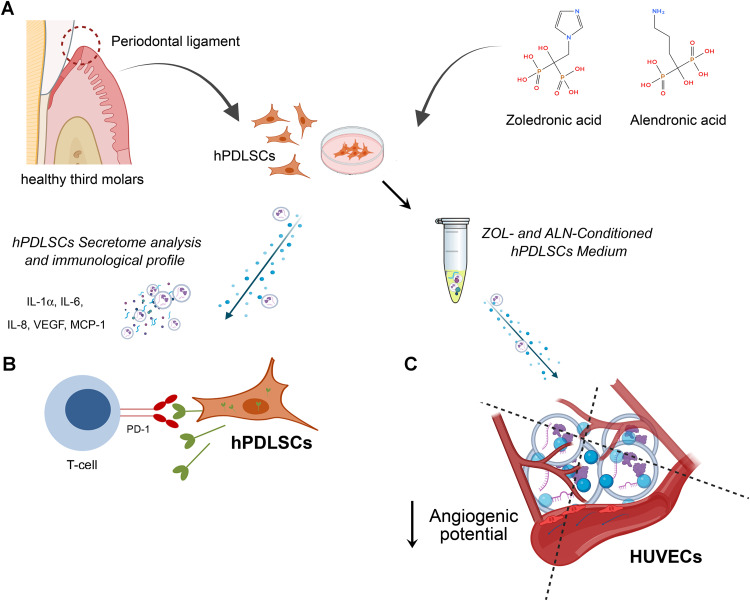
Schematic representation of the study design and the main findings. (A) hPDLSCs were isolated and cultured in the presence of increasing concentrations of ZOL or ALN. (B) hPDLSCs exposed to ZOL or ALN were analyzed for cytokines secretion and PD-L1 expression. (C) The exposure of HUVECs to ZOL- and ALN-conditioned hPDLSCs medium reduced their ability to form capillary-like tubes.

In conclusion, this is the first study describing the effects of CM from ZOL- or ALN-treated hPDLSCs on angiogenesis *in vitro* (Fig 7). Our findings suggest that neither ZOL or ALN treatment induces significant alterations of IL-1α, IL-6, IL-8, VEGF and MCP-1 in hPDLSCs secretome. Nevertheless, the exposure to ZOL ≥ 1.5 μM can induce a pro-inflammatory phenotype in hPDLSCs via a down-regulation of PD-L1 expression. Additionally, preconditioning with ZOL ≥ 1.5 μM leads to impaired endothelial migration and tubulogenic activity, accounting for a delayed repair of mucosal lesions. ALN influences inflammatory phenotype of hPDLSCs at all tested concentrations. The reduced expression of nuclear and surface PD-L1 in hPDLSCs exposed to ALN 2 μM indicates a proinflammatory action. Conversely, the reduced nuclear PD-L1 and increased surface PD-L1 at the highest ALN concentrations (10 μM) suggest a different function of PD-L1 based on its subcellular localization. Moreover, ALN-preconditioning impairs angiogenic potential of hPDLSCs secretome, by reducing endothelial cells migration and tubulogenic activity. Despite the concurrent reduction in angiogenic potential and PD-L1 expression is compelling, further studies are needed to establish a causal association between PD-L1 expression and the angiogenic potential of hPDLSCs secretome. Overall, our preliminary findings indicate that the exposure to ZOL and ALN may drive a shift towards a pro-inflammatory phenotype in hPDLSCs, according to the reduced PD-L1 expression. Moreover, the angiogenic properties of hPDLSCs were compromised following exposure to ZOL and ALN. Additional studies will be necessary to determine whether the antiangiogenic effects of BPs observed in vitro are, at least in part, mediated by the pro-inflammatory phenotype of hPDLSCs.

Moreover, in the realm of periodontal regenerative medicine, our research underscores the importance of optimizing the angiogenic potential of hPDLSCs for enhanced tissue repair and regeneration. Future advancements may focus on refining culture conditions or preconditioning regimens to counteract the negative effects of BPs. Such improvements could facilitate the successful integration of transplanted stem cells, to ensure their functionality and promoting long-term tissue healing and regeneration.

## Supporting information

S1 Fig Uncropped raw images used in Fig 2. (PDF)

S1 File Data used to build graphs and figures. (XLSX)
