# Supplementary material for: The role of amino bisphosphonates zoledronate and alendronate in shaping immunomodulatory profile and angiogenic potential of human periodontal ligament stem cells
Source: PLoS One. 2025 Dec 1;20(12):e0335744. doi: 10.1371/journal.pone.0335744 (PMC12668531; doi:10.1371/journal.pone.0335744)

Original images for PD-L1 and GAPDH determination

1st replicate

PD-L1 determination in hPDLSCs exposed to ZOL or ALN

1st Exposure PD-L1 (Figure S1)

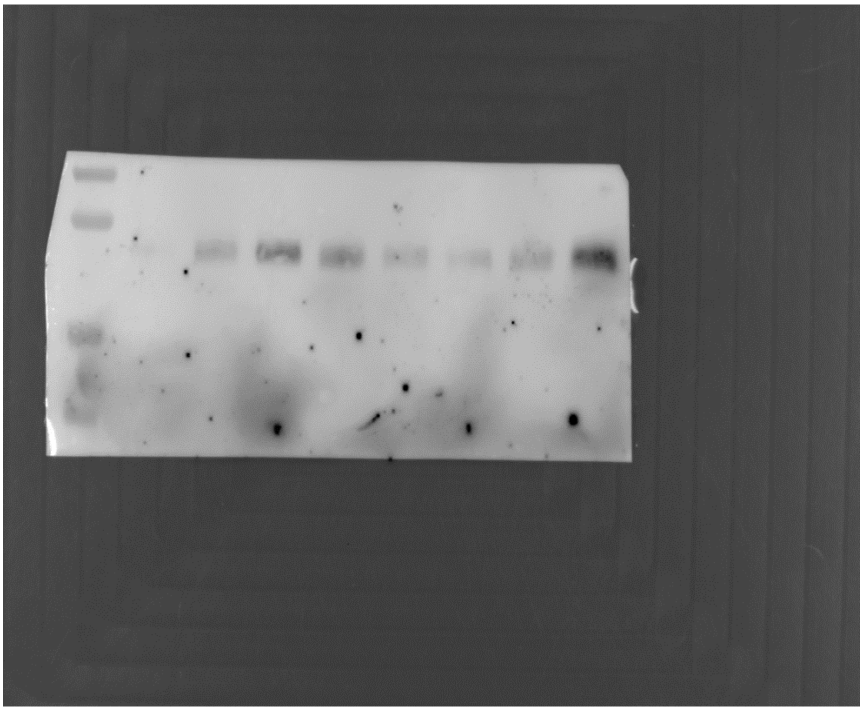

With labeling (Figure S1)

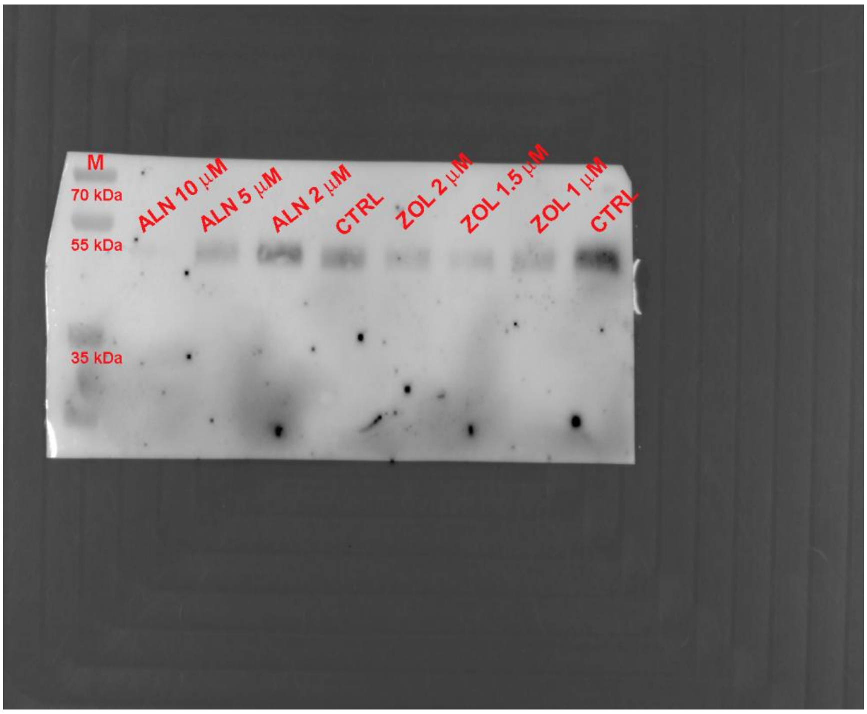

2nd Exposure PD-L1 (Figure S2)

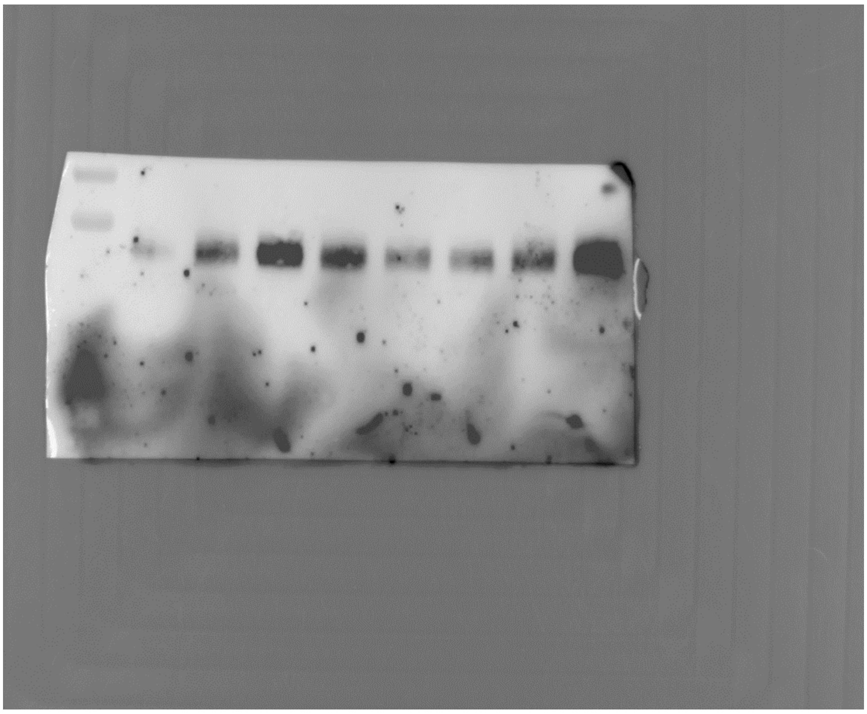

With labeling (Figure S2)

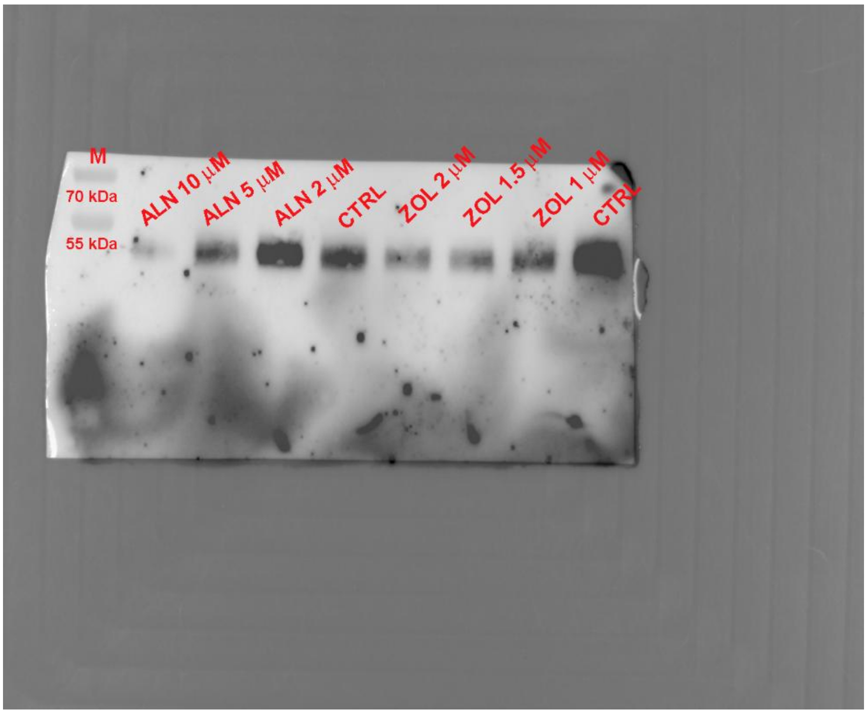

GAPDH determination in hPDLSCs exposed to ZOL or ALN

1st Exposure GAPDH (Figure S3)

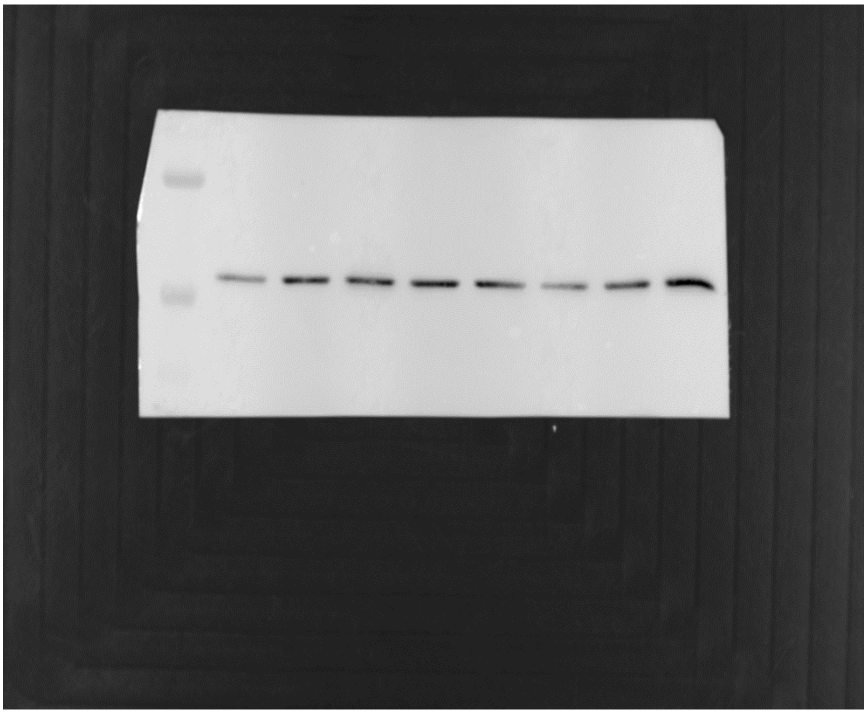

With labeling (Figure S3)

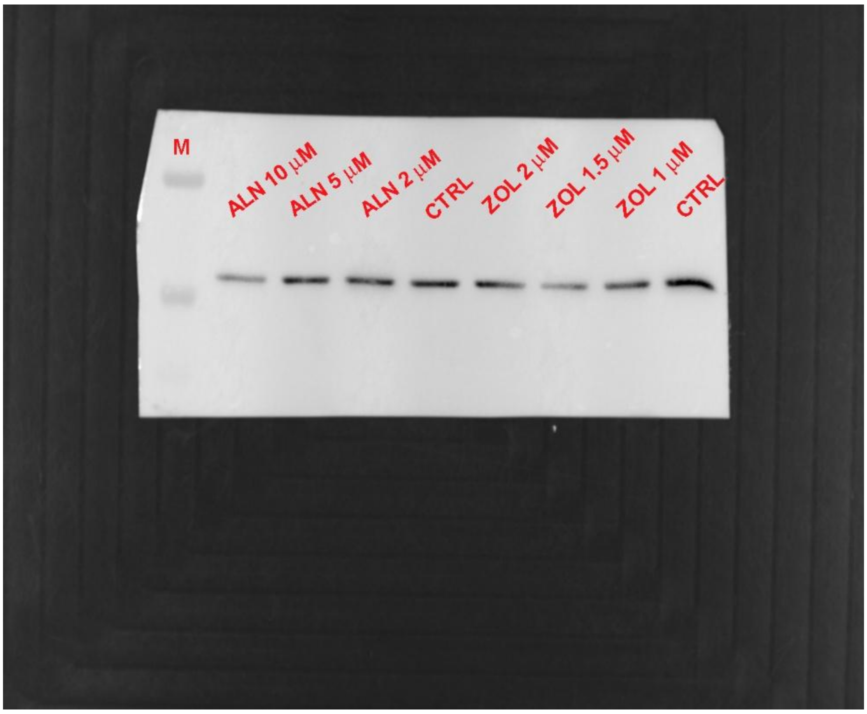

2nd Exposure GAPDH (Figure S4)

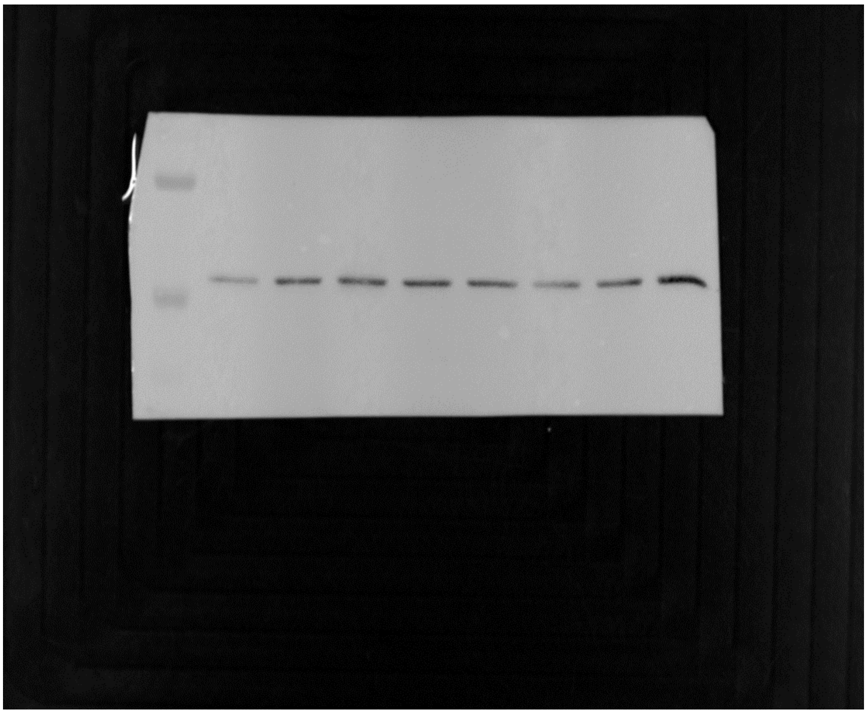

With labeling (Figure S4)

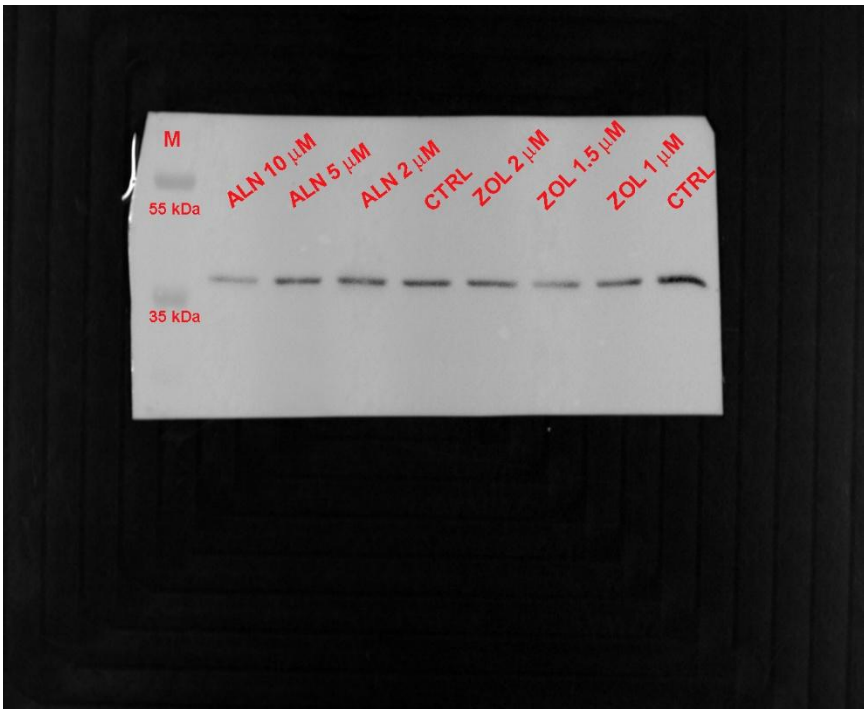

2st replicate

PD-L1 determination in hPDLSCs exposed to ZOL or ALN

PD-L1 (Figure S5)

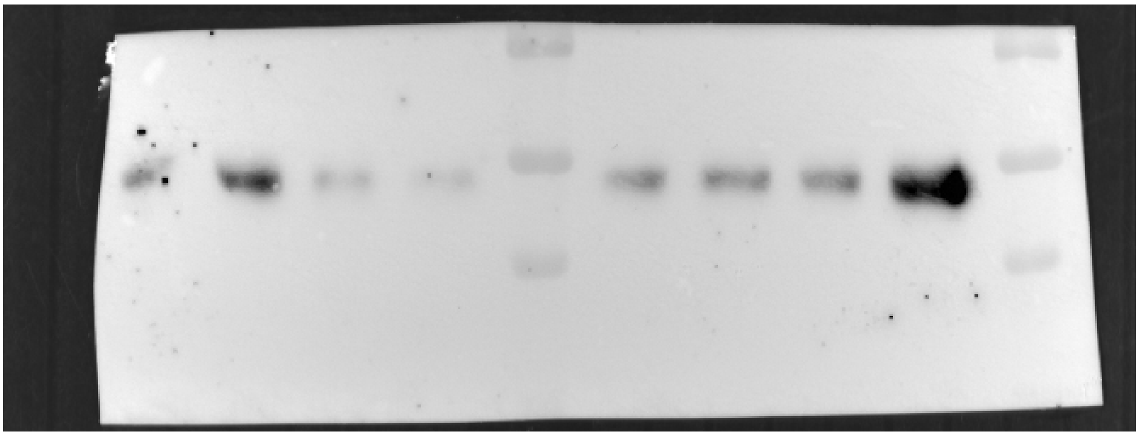

With labeling (Figure S5)

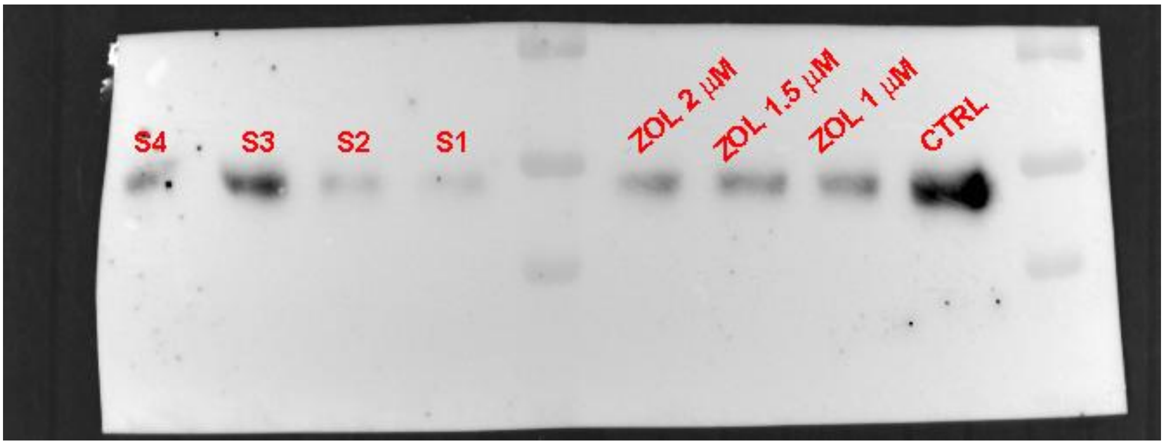

GAPDH (Figure S6)

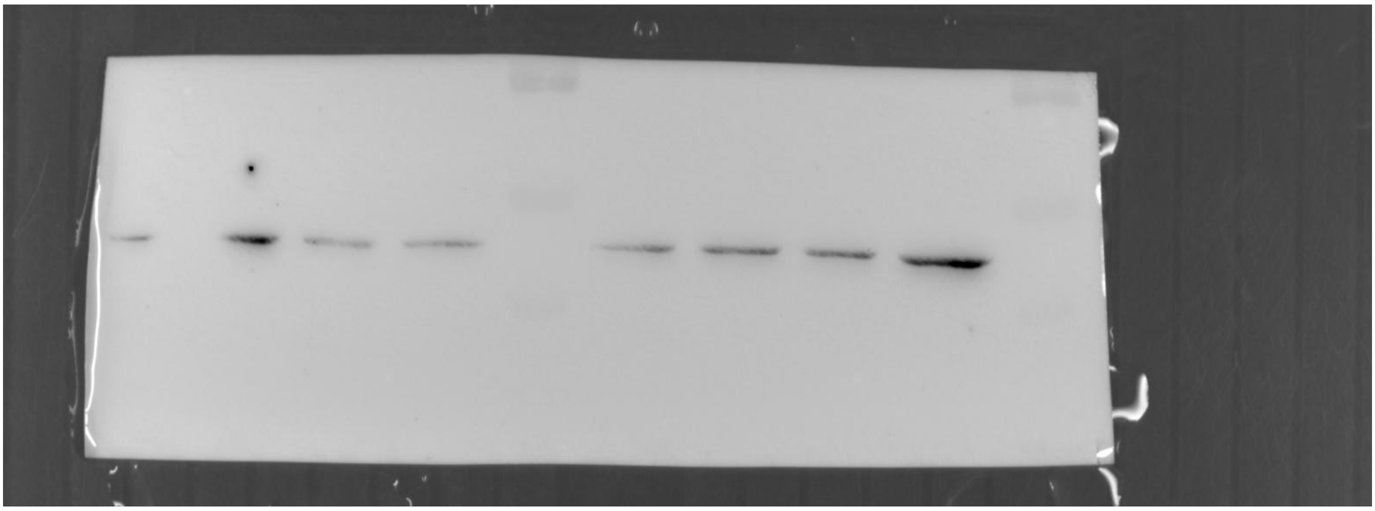

With labeling (Figure S6)

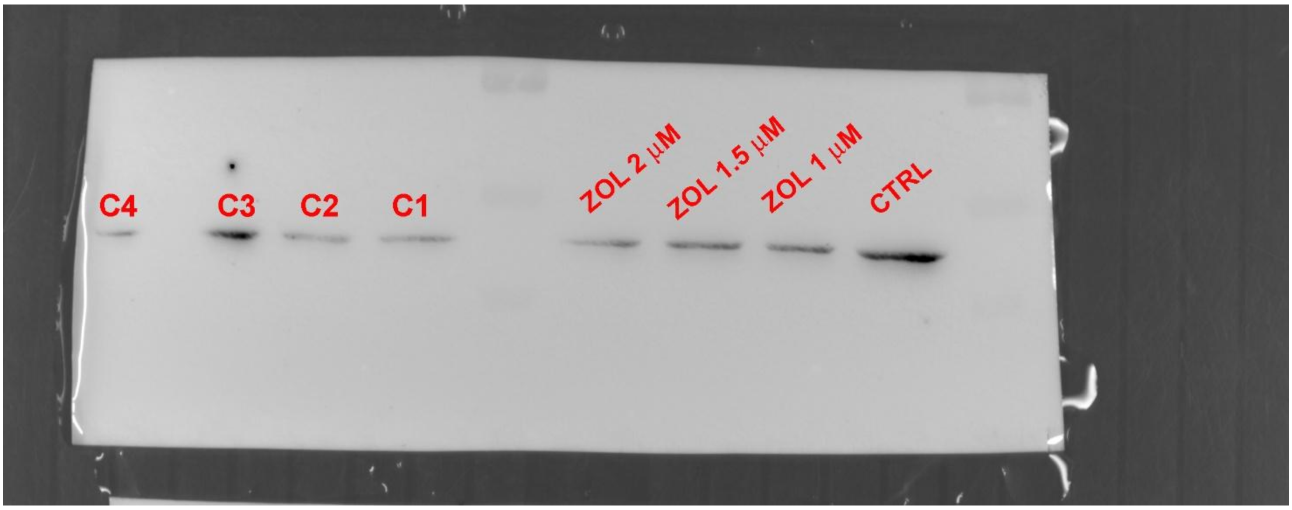

PD-L1 (Figure S7)

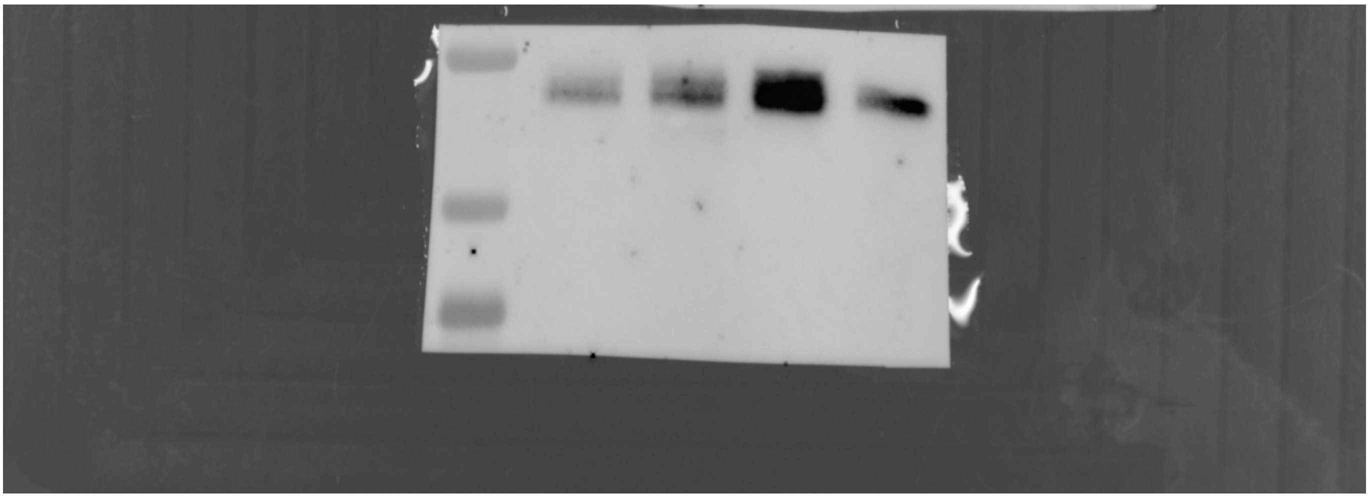

With labeling (Figure S7)

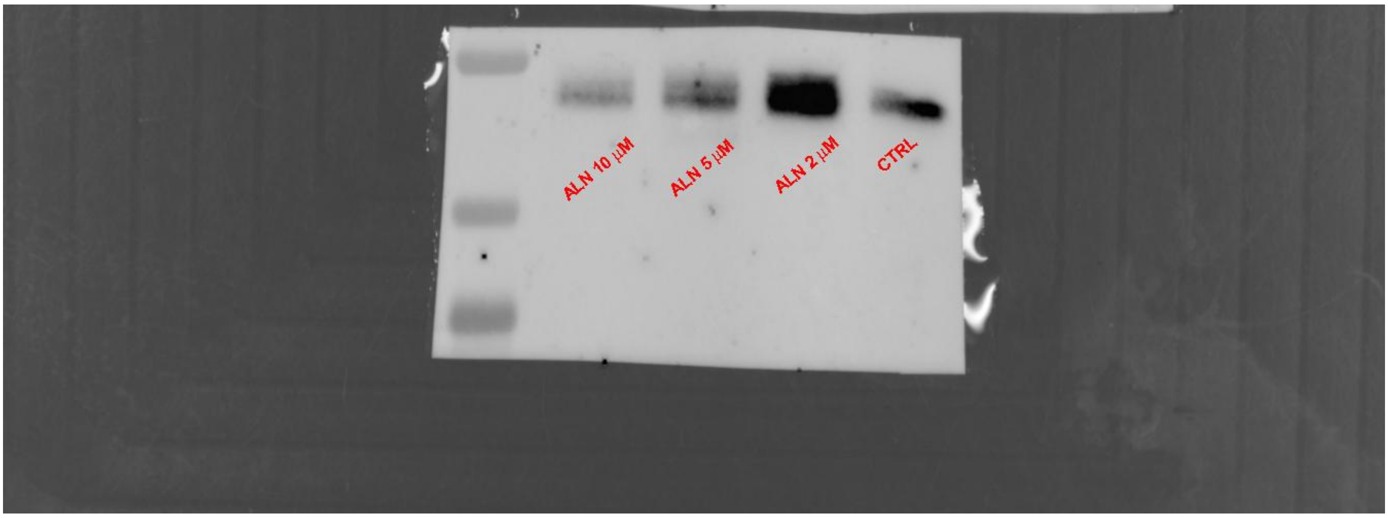

GAPDH (Figure S8)

Please note that the membrane has been rotated by 180 °C

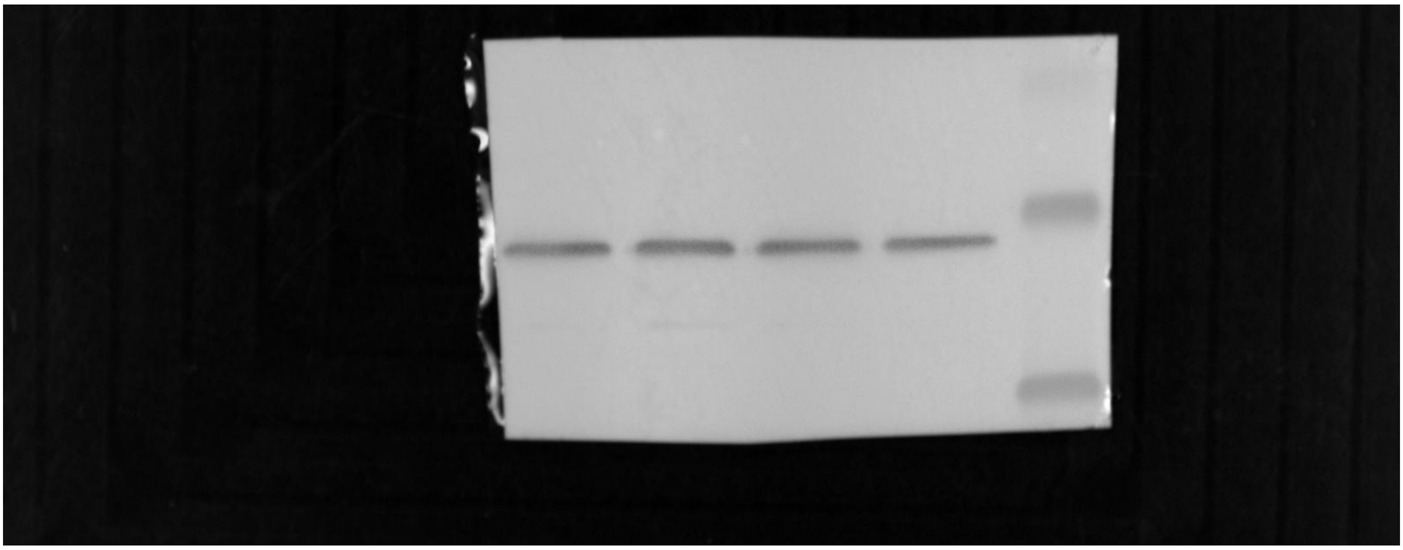

With labeling (Figure S8)

Please note that the membrane has been rotated by 180 °C

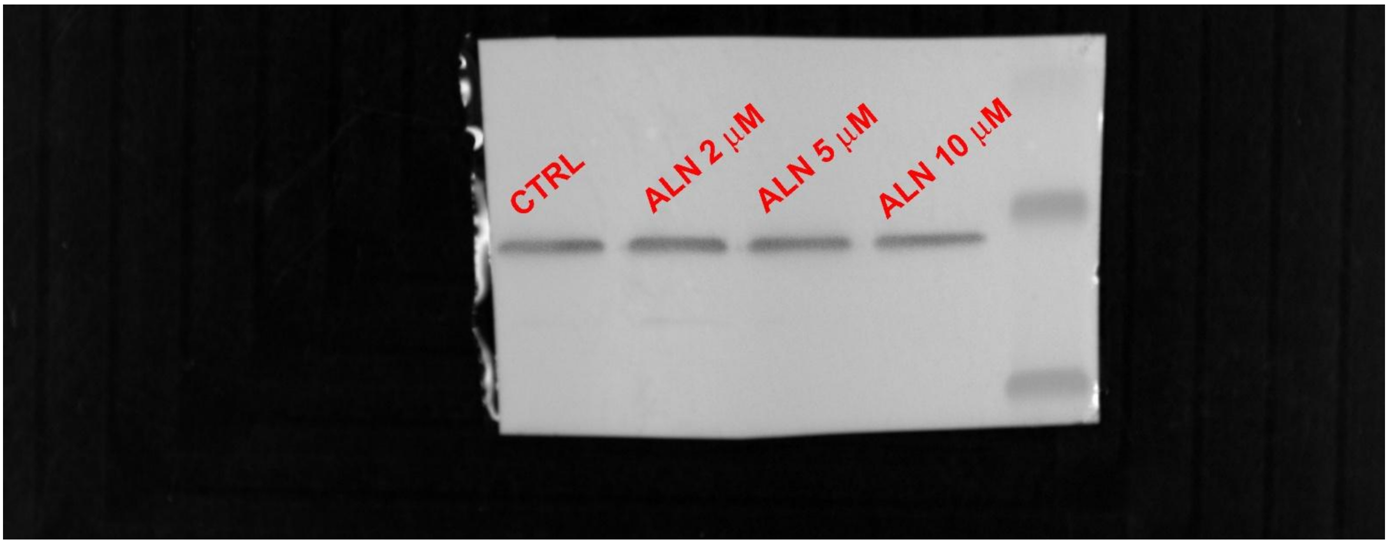

Supplement: S1 Fig — (PDF) [file pone.0335744.s001.pdf]
